# Implementing antibody-drug conjugates (ADCs) in HER2-positive breast cancer: state of the art and future directions

**DOI:** 10.1186/s13058-021-01459-y

**Published:** 2021-08-11

**Authors:** Emanuela Ferraro, Joshua Z. Drago, Shanu Modi

**Affiliations:** 1 Department of Medicine, Memorial Sloan Kettering Cancer Center, New York, NY USA; 2 Weil Cornell Medical College, New York, NY USA

**Keywords:** Antibody-drug conjugate, ADCs, HER2-positive breast cancer, Anti-HER2 ADCs, Anti-HER2 antibody conjugate, Novel anti-HER2 ADCs

## Abstract

The development of anti-HER2 agents has been one of the most meaningful advancements in the management of metastatic breast cancer, significantly improving survival outcomes. Despite the efficacy of anti-HER2 monoclonal antibodies, concurrent chemotherapy is still needed to maximize response. Antibody-drug conjugates (ADCs) are a class of therapeutics that combines an antigen-specific antibody backbone with a potent cytotoxic payload, resulting in an improved therapeutic index. Two anti-HER2 ADCs have been approved by the FDA with different indications in HER2-positive breast cancer. Ado-trastuzumab emtansine (T-DM1) was the first-in-class HER2-targeting ADC, initially approved in 2013 for metastatic patients who previously received trastuzumab and a taxane, and the label was expanded in 2019 to include adjuvant treatment of high-risk patients with residual disease after neoadjuvant taxane and trastuzumab-based therapy. In 2020, trastuzumab deruxtecan (T-DXd) was the second approved ADC for patients who had received at least 2 lines of anti-HER2-based therapy in the metastatic setting. The success of these two agents has transformed the treatment of HER2-positive breast cancer and has re-energized the field of ADC development. Given their advanced pharmaceutical properties, next-generation HER2-targeted ADCs have the potential to be active beyond traditional HER2-positive breast cancer and may be effective in cells with low expression of HER2 or *ERBB2* mutations, opening a spectrum of new possible clinical applications. Ongoing challenges include improving target-specificity, optimizing the toxicity profile, and identifying biomarkers for patient selection. The aim of this review is to summarize the principal molecular, clinical, and safety characteristics of approved and experimental anti-HER2 ADCs, contextualizing the current and future landscape of drug development.

## Introduction

Historically, HER2-positive breast cancer (BC) has been recognized to have a poor prognosis, with a median overall survival of 15 months with traditional chemotherapy treatments in the metastatic setting [1]. Trastuzumab, the first HER2-targeting monoclonal antibody (mAB) developed, represents one of the most significant advancements in the management of solid tumors. Today, there are 8 approved HER2 targeted agents and the median survival is over 5 years for patients with advanced-stage disease, though it may be even longer for patients who receive novel therapies [2]. The maximal antitumoral activity of anti-HER2 agents is achieved in combination with chemotherapy, and this effect may be related to the heterogeneity of HER2 expression among other mechanisms of primary resistance [3]. Additionally, HER2 overexpressing cells have high proliferation rates, resulting in higher responsiveness to cytotoxic therapies.

Based on the synergistic effects of HER2-inhibition and chemotherapy, a new clas of drug have been developed. ADCs combine the antitumoral properties of both of these approches in a single pharmacological entity. The aim of this review is to provide a summary of the clinical data on ADCs approved by the US Food and Drug Administration (FDA), as well as novel ADCs under development for the treatment of HER2-positive breast cancer.

## Pharmacodynamic of ADCs

ADCs consist of a humanized mAB, mainly immunoglobulin G (IgG), bound to a cytotoxic agent, called the payload, via a molecular linker. Canonically, the antibody component, through the fragment antigen-binding (Fab) portion, recognizes and binds its target antigen on the cell surface, inducing internalization of the entire ADC complex via endocytosis. Cleavage of the linker in the lysosomes through proteolysis and/or acidification results in the release of the payload portion inside the cancer cells [4]. This target-dependent activation allows for selective cytotoxicity against cancer cells, resulting in a significant reduction of systemic side effects. The benefits of an ADC approach, however, may be more complex based on the properties of the individual components. For example, membrane permeable payloads, once released in the cell, can diffuse into the adjacent and neighboring cells where they can induce a non-antigen-dependent cytotoxicity, called the bystander effect. The bystander effect felt to be responsible for the broad activity of some ADCs in cancers with low or heterogeneous expression of the target antigen. Several other mechanisms mediated by the fragment crystallizable (Fc) region of the mAB may be involved in the anti-tumoral activity of ADCs via antibody-dependent cellular cytotoxicity (ADCC) [4]. Moreover, ADCs targeting oncogenes like HER2 can preserve a trastuzumab-like activity inhibiting HER2 dimerization and activation of the downstream pathways.

While the ADC target is often emphasized, the anti-tumor activity and toxicities of ADCs are intricately tied to the properties of the linker and the payload. The aim of the linker is to ensure the payload remains attached to the antibody backbone in the bloodstream and non-cancer compartment, avoiding the premature release of the cytotoxic drug. The linkers are classified as being either cleavable or non-cleavable, based on their biochemical properties. Cleavable linkers can release the payload in response to conditions such as the presence of proteases, redox environments, or acidic pH in tumor cells or the microenvironment. Non-cleavable linkers are broken down inside the cancer cells by proteasomal degradation. The payloads are potent chemotherapy agents that belong to distinct drugs classes including microtubule disrupters, DNA intercalators, and topoisomerase 1 inhibitors. It is estimated that only 0.1% of the delivered ADC reaches the tumoral area, on average, indicating that effective payloads can induce cytotoxicity at sub-nanomolar concentrations [5]. An additional factor influencing the overall potency and therapeutic index of ADCs is the drug to antibody ratio (DAR), specifically the average number of cytotoxic molecules linked to each antibody [6].

## Current FDA-approved ADCs for HER2-positive breast cancer

In this section, we discuss the most relevant evidence that led to the approval of the first ADCs in HER2-positive breast cancer. Investigational uses will be discussed in the following sections.

### Trastuzumab emtansine

Trastuzumab emtansine (T-DM1) was the first HER2-targeting ADC approved for the treatment of advanced HER2-positive BC and more recently approved for early-stage high-risk patients with residual disease after neoadjuvant treatment. T-DM1 contains the backbone of trastuzumab linked to mertansine (DM1), a potent microtubule inhibitor, via a non-cleavable thioether linker (N-maleimidomethyl), with a DAR of 3.5. All the cytotoxic functions of trastuzumab, including ADCC and signaling inhibition, are preserved, in addition to the antitumoral effects of the payload [4].

Clinically, the indication for T-DM1 in the metastatic setting is as a second-line treatment and beyond, based on data from the pivotal trials EMILIA and TH3RESA. The EMILIA was a phase III study that investigated the efficacy of T-DM1 versus capecitabine and lapatinib in patients with HER2-positive BC progressing on trastuzumab and taxane therapy (*n* = 911 patients). The results favored T-DM1, showing an improvement in overall survival (OS) (29.9 vs 25.9 months; HR 0.75, 95% CI 0.64–0.88), after a median follow-up of 47.8 months [7].

Similarly, the benefits and superior efficacy of T-DM1 compared to an established standard of care treatments was confirmed in patients with more heavily pre-treated HER2-positive metastatic breast cancer (MBC), as tested in the TH3RESA trial: progression-free survival (PFS) was 6.2 months and 3.3 months in T-DM1 arms compared to physician’s choice of therapy (HR 0.53, 95% CI 0.42–0.66; *P* < 0.0001) [8], and OS was 22.7 versus 15.8 months, respectively [9].

More recently the benefits of T-DM1 have been extended to include patients in the early stage with residual disease after neoadjuvant treatment, recognized as a group at high risk of recurrence, based on the results of the KATHERINE trial [10]. The 3-year invasive disease-free survival (IDFS) was significantly higher in the T-DM1 group than in the control arm treated with trastuzumab alone, with a hazard ratio of 0.5 (88.3 vs 77%, in T-DM1 and trastuzumab arms, 95% confidence interval, 0.39 to 0.64; *P* < 0.001). Investigations into using T-DM1 to replace trastuzumab plus chemotherapy in early-stage HER2-positive BC have not produced definitive results [11], and this approach is still under investigation (NCT03587740). Of interest, T-DM1 seems to be active in cancers with HER2 mutations, regardless of amplification/expression status—providing an opportunity to expand in a setting of high unmet need [12]. Overall, T-DM1 is associated with manageable adverse events including gastrointestinal toxicity and neuropathy and left ventricular ejection fraction (LVEF) decline, mostly grade 1/2 in severity. Thrombocytopenia, the dose-limiting toxicity, along with an increase in liver enzymes, including risk of liver failure, are the most frequent high-grade toxicities as reported in clinical trials and real-world experience [7, 13].

### Trastuzumab deruxtecan

Trastuzumab deruxtecan (T-DXd; previously called DS8201a) is the second FDA-approved ADC for the management of advanced-stage HER2-positive BC treated with at least two prior lines of HER2-targeting therapy. Like T-DM1, it is composed of a mAb backbone of trastuzumab however there are many pharmaceutical differences between the two ADCs. T-DXd has a DAR of 8 (compared T-DM1, which has a DAR of 3.5) and its cytotoxic payload, derived from exatecan, is a potent topoisomerase I inhibitor rather than a microtubule inhibitor. Additionally, T-Dxd incorporates a cleavable linker, thought to be acted upon by cathepsins, lysosomal enzymes which are upregulated in many cancer cells. Relatedly, the payload is membrane permeable, hence capable of exerting the bystander effect, theoretically allowing activity even in tumors with heterogeneous or low expression of HER2. This property is not observed with T-DM1. All these features may explain the anticancer activity of T-DXd in tumors that are refractory to T-DM1.

In the first-in-human phase 1 study of T-DXd, which included 111 patients with heavily pretreated advanced HER2-positive breast cancer, T-DXd monotherapy exhibited a response rate of 59.5% (95% CI, 49.7 to 68.7), with a median duration of response of 20.7 months [14]. Based on these results, the phase 2 registration study, DESTINY-Breast01 was initiated, designed for patients with HER2-positive MBC having received prior treatment with T-DM1. At a median follow-up of 11.1 months, in a cohort of 184 patients, the overall response rate was 60.9% (95% CI, 53.4 to 68.0), of which 6% experienced a complete response (CR) and an overall disease-control rate (DCR) of 97.3% (95% CI, 93.8 to 99.1) [15]. Interestingly, the median time to achieve a response was 1.6 months (95% CI, 1.4 to 2.6), suggesting rapid anticancer activity similar to classical chemotherapeutic agents. The most recently presented update, now with a median of 20+ months of follow-up, showed a median duration of response (DOR) of 20.8 months, median PFS of 19.4 months, and an interim median OS of 24.6 months [16]. These results were observed in a heavily pre-treated population of HER2-positive MBC patients with a median of 6 lines of prior therapy and ultimately led to a breakthrough therapy designation in the US and an accelerated approval in December 2019. Phase 3 trials results in 2° and 3° line are awaited. The activity of T-DXd in earlier lines is now being investigated in multiple studies (Table 1).

**Table 1:** Ongoing clinical trials with T-Dxd including breast cancer patients

NCT identifier	Trial name	Phase	Experimental arm	Control arm	Setting	Population	Primary endpoint
NCT03523585	DESTINY-Breast02	III	T-DXd	Investigator’s choice	Prior T-DM1	HER2+ MBC	PFS
NCT03529110	DESTINY-Breast03	III	T-DXd	T-DM1	Prior taxane +trastuzumab	HER2+ MBC	PFS
NCT03734029	DESTINY-Breast04	III	T-DXd	Investigator’s choice	1 or 2 prior chemotherapies. POD on ET if HR+	HER2-low MBC	PFS
NCT04132960	DAISY	II	T-DXd	-	≥ 2^nd^ line	MBC C1: HER2 over-expressing C2: HER2 low expressing C3: non expressing ^d^	BOR
NCT04752059	TUXEDO-1	II	T-DXd	-	≥ 2nd line	Newly diagnosed or progressing BM HER2+MBC	CNS ORR
NCT04784715	DESTINY-Breast09	III	T-DXd+/− P	THP	1st line	No prior chemo or POD ≤ 6 mo from adj therapy HER2+ MBC	PFS
NCT04622319	DESTINY-Breast05	III	T-DXd	T-DM1	Post-neoadjuvant	Primary BC with residual invasive disease	IDFS
NCT04420598	DEBBRAH	II	T-DXd	-	≥ 2nd line	BM or LMD in HER2+or HER2-low MBC	CNS ORR, 16w PFS and OS of T-DXd
NCT04494425	DESTINY-Breast06	III	T-DXd	treatment of Investigator’s Choice	≥ 3rd line	HR+ HER2-low MBC	PFS
NCT04257162	HER2-PREDICT	-	T-DXd	-	-	Patients treated with T-DXd+	ERBB2 mRNA cut-point predictive of T-DXd response
NCT04042701	-	Ib/II	T-DXd+ pembrolizumab	-	≥ 2nd line	HER2+ or low MBC and HER2+ or mut NSCLC	DLT and ORR
NCT04553770	-	II	T-DXd+/− ANA	-	Neoadjuvant setting	HR+ HER2-low EBC	pCR rate
NCT04556773	DESTINY-Breast 08	Ib	T-DXd+ Durva or T or Capi or ANA or Fulv or Cape	-	≥ 2nd line	HER2-low MBC	Safety
NCT04294628	-	I	T-DXd	-	≥ 2nd line	Any advanced solid tumor, HER2+	Effect of T-DXd on Top1cc
NCT04539938	HER2CLIMB-04	II	T-DXd+ tucatinib	-	≥ 2nd line	HER2+MBC	ORR
NCT04704661	DASH	I/Ib	T-DXd and AZD6738^a^	-	≥ 2nd line	HER2+/low/ mutant solid tumors	DLT and RP2D
NCT03742102	BEGONIA	Ib/II	Durvalumab + T-DXd or Capi or Ole^b^ or T or DS-1062^c^	-	1st line	TNBC	Safety and ORR

*Abbreviations: MBC* metastatic breast cancer, *EBC* early breast cancer, *PFS* progression-free survival, *BOR* best objective response rate, *C* cohort, *mo* months, *BM* brain metastases, *P* pertuzumab, *THP* taxane, trastuzumab, and pertuzumab, *IDFS* invasive disease-free survival, *CNS* central nervous system, *ORR* overall response rate, *OS* overall survival, *DLT* dose-limiting toxicities, *mut* mutant *NSCLC*, non-small cell lung cancer, *pCR* pathological complete response, *ANA* anastrozole, *Durva* durvalumab, *T* paclitaxel, *Capi* capivarsetib, *Fulv* fulvestrant, *Cape* capecitabine, *Top1cc* topoisomerase 1 cleavable complex, *RP2D* recommended phase 2 dose, *Ole* oleclumab, *C* cohort, *LMD* leptomeningeal disease

^a^AZD6738: ATR inhibitor

^b^Oleclumab: anti-CD73 Ab

^c^ADC anti-TROP2

^d^HER2 over-expressing: HER2 IHC3+ or HER2 IHC2+/ISH+; HER2 low-expressing IHC1+ or IHC2+/ISH−; HER2 non-expressing IHC0

Note: https://clinicaltrials.gov/ April 2021

The most common toxicities associated with T-Dxd include nausea and bone marrow suppression and are largely grade 1/2 in nature; however, there is also an important risk of lung toxicity, broadly defined under the umbrella term of interstitial lung disease (ILD), which ranges in severity and is discussed further in the “Toxicity profile” section.

## ADCs under investigation

A number of next-generation HER2-targeted ADCs are currently under investigation in clinical trials. These novel agents have been designed with different payloads and linker technologies in order to further improve upon their efficacy and tolerability. The main pharmacological properties of these next-generation anti-HER2 ADCs are summarized in Table 2.

**Table 2:** Pharmacological properties of anti-HER2 ADCs undergoing clinical trials

ADC name	Ab	Linker	Payload	DAR	Ongoing clinical trials^**a**^
SYD985	Trastuzumab	Valine-citrulline linker (cleavable)	Seco-DUBA (DNA-alkylating)	2.8	NCT03262935 NCT04602117 NCT04235101 NCT01042379
ZW49	ZW25	Cleavable	N-acyl sulfonamide auristatin (microtubule inhibitor)	N.A.	NCT03821233
PF-06804103	Trastuzumab-derived Ab	Maleimidocaproyl-valine-citrulline linker (cleavable)	Aur-06380101	4	NCT03284723
MRG002	Anti-HER2 IgG1	N.A.	Monomethyl auristatin E	NA	CTR20181778 NCT04492488, NCT04742153
GQ1001	Anti-HER2 IgG	NA	NA	NA	NCT04450732
ARX788	Modified heavy chain Ala114 of anti-HER2 mAb	Amberstatin (AS269) (non-cleavable)	Dolastatin monomethyl auristatin F (microtubule inhibitor)	1.9	NCT03255070
A166	Trastuzumab	Valine citrulline peptide (cleavable linker)	Duostatin-5 (microtubule inhibitor)	N.A.	NCT03602079
XMT-1522	HT-19 (anti-HER2 IgG1)	Cleavable hydrophilic polymer	AF-HPA (microtubule inhibitor)	12	NCT02952729
RC48-ADC	Hertuzumab (anti-HER2 humanized Ab)	Valine citrulline peptide (cleavable linker)	Monomethyl auristatin E (microtubule inhibitor)	4	NCT04329429 NCT04280341 NCT04311034 NCT04714190 NCT04073602 NCT04264936 NCT03556345 NCT03500380 NCT03052634
BDC-1001	Trastuzumab	Non cleavable linker	TLR7/8 inhibitor	NA	NCT04278144
FS-1502	Trastuzumab	NA	Monomethyl Auristatin F	NA	NCT03944499
GQ1001	NA	NA	NA	NA	NCT04450732
ALT-P7	Trastuzumab biobetter HM2	Cysteine-containing peptide	Monomethyl auristatin E (microtubule inhibitor)	NA	NCT03281824

*Abbreviations*: *AF-HPA* auristatin F-hydroxypropylamide, *NA* non- available

^a^https://clinicaltrials.gov/ April 2021

### Trastuzumab duocarmazine

SYD985 (or [vic]-trastuzumab duocarmazine) is a HER2 ADC consisting of a backbone of trastuzumab conjugated with a cleavable linker and a duocarmycin payload (a potent DNA alkylating molecule, incorporated as its inactive prodrug form, *seco*-duocarmycin). The payload in this context is membrane-permeable and hence also has the potential to enter neighboring cells regardless of HER2 expression [17]. The phase 1 study of this ADC showed an acceptable toxicity profile and signals of anticancer activity in HER2-positive and “HER2-low” breast cancers, defined as HER2 1+ or 2+/FISH non-amplified. In the expansion arm of this study for patients with HER2-positive MBC, 16/48 (33%, 95% CI 20.4–48.4) achieved an objective response [18]. These results formed the basis of the phase III TULIP trial (NCT03262935), enrolling patients with HER2-positive MBC pretreated with T-DM1 and randomizing them to either trastuzumab duocarmazine versus treatment of physician’s choice, with a primary endpoint to evaluate PFS. Results have not yet been reported. SYD985 is currently also under investigation in combination with paclitaxel (NCT04602117) and with niraparib (a PARP inhibitor) (NCT04235101). A combination of SYD985 with Adriamycin and Cyclophosphamide is also being tested in the I-SPY trial (NCT01042379), a large adaptive neoadjuvant trial designed to evaluate the pathological complete response (pCR) rate with different combinations of biological agents plus chemotherapy. The preclinical and clinical data in support of SYD985 in HER2-low BC are discussed below.

### A166

A166 is an ADC comprised of trastuzumab linked to duostatin-5 (an auristatin derivative) as the payload. The first efficacy results from a phase I trial of 27 evaluable patients were presented in 2020 with a DCR of 59%, with partial responses observed in 7 patients (26%) at the dose levels of 3.6 mg/kg and 4.8 mg/kg [19]. This study is ongoing and updated results are awaited (NCT03602079).

### XMT-1522

XMT-1522 is anti-HER2 ADC containing HT-19, a human IgG1 anti-HER2 mAB that binds to domain IV of HER2 to an epitope that is distinct from the trastuzumab-binding site, with a payload that is an auristatin derivative (AF-HPA). Preclinical data reveal XMT-1522 is effective in HER2-positive BC and gastric cancer cell lines and xenograft models resistant to T-DM1 [20]. Preliminary results of a phase I study showed an overall DCR of 83% (seen in 5/6 patients) with 1 PR, all seen at doses of either 16 or 21.3 mg/m2; a DCR of 25% (3/12) has been reported in patients treated at doses less than 16 mg/m2 (NCT02952729) [21].

### RC48-ADC

RC48 is a novel anti-HER2 ADC which includes hertuzumab (a new anti-HER2 mAB) conjugated with monomethyl auristatin E (MMAE) via a cleavable linker. In a phase I study, RC48 has shown an acceptable toxicity profile and promising antitumor activity in solid tumors with a reported objective response rate (ORR) of 33.3% and DCR of 53% for patients treated at either the 2.0 and 2.5 mg/kg cohorts [22]. A phase Ib study has evaluated the activity of RC48-ADC in HER2-positive MBC. Among 30 evaluable patients, disease-control was observed in 29/30 (96.7%) patients, comprised of 11 partial response (PR) and 18 cases of stable disease (SD), for an ORR of 26.7% and 46.7% in the 1.5 mg/kg and 2.0 mg/kg cohorts, respectively (57.1% in trastuzumab-naive patients and 33.3% in patients pretreated with trastuzumab) [23].

### ALT-P7 (HM2/MMAE)

ALT-P7 is a novel HER2-targeting ADC composed of trastuzumab variant conjugated to molecules of monomethyl auristatin E (MMAE). Primary results from the first in human study showed a DCR of 77.3% (17/22) with PRs in 2/15 patients with measurable lesions. The median PFS at doses from 2.4 to 4.8 mg/kg was 6.2 months (95% CI 2.5–9.9 months) in a population with a median of 6 previous treatment lines [24]. A phase 2 study is planned.

### ARX788

ARX788 is a site-specific ADC that consists of an anti-HER2 antibody linked to a highly potent tubulin inhibitor AS269, using a unique non-natural amino acid-enabled conjugation technology and a non-cleavable linker [25]. First results from a phase 1 trial showed antitumor activity in HER2-positive BC leading to a fast track designation granted by the FDA in January 2021 [26]. Among 48 evaluable patients, the ORR was 56% at 1.3 mg/kg and further increased to 63% at 1.5 mg/kg. A 2-part phase 1 dose-escalation trial is currently ongoing in patients with HER2-positive solid tumors (NCT03255070).

### PF-06804103

PF-06804103 is an ADC that includes three components: a trastuzumab-derived antibody, AUR-06380101 (a novel potent auristatin derivate), and an enzymatically cleavable linker. PF-06804103 has shown efficacy in low HER2-expressing breast, gastric, and lung tumor models [27]. Preliminary results of a dose-escalating phase 1 study, including patients with HER2-positive breast and gastroesophageal progressing on several lines of prior treatment, revealed an ORR of 52.4% in the patients treated with doses ≥3 mg/kg (11/21 patients). The median number of prior therapies was 6 (3-18) and all patients had prior HER2-targeted therapy [28]. Final results are awaited (NCT03284723).

## ADCs in pre-clinical development

Multiple additional novel agents have shown encouraging results in preclinical studies that support their future clinical development for patients with HER2-positive solid tumors, including breast cancer. Here we highlight just a few of these candidates. MRG002 and ZW49 are two ADCs that use auristatin as a payload conjugated to different mAbs, the former with a humanized anti-HER2 IgG1 monoclonal antibody and the latter using ZW25, an anti-HER2 biparatopic antibody recognizing the binding domains of trastuzumab and pertuzumab, respectively [29, 30]. Phase 1 trials of both ADCs are currently ongoing (CTR20181778, NCT04492488, NCT04742153, NCT03821233). Lastly, BDC-1001 is composed of a biosimilar of trastuzumab chemically conjugated to a TLR 7/8 agonist with an intervening non-cleavable linker. BDC-1001 is able to activate antigen-presenting cells, while retaining antibody-mediated effector functions such as ADCC. Preclinical data show that BDC-1001 induces a potent immune-mediated antitumor effect in xenograft models [31] and a first in human study has demonstrated safety [32]. Efficacy results of BDC-1001 alone or in combination with anti-PD1 are still awaited (NCT04278144). Finally, a newly engineered pertuzumab-based ADC with less affinity for HER2 at acidic endosomal pH showed an increased lysosomal delivery and cytotoxicity in a HER2-low-expressing xenograft models and is expected to enter further clinical testing [33].

## Activity of ADCs in HER2-low breast cancer

The technological advancements and resulting efficacy of next-generation ADCs have raised new questions about the historical paradigm which posits dichotomous classification of HER2 breast cancers as either positive or negative. According to the standard definitions per the 2018 ASCO and College of American Pathologists guidelines [34], breast tumors with low expression of HER2, defined as immunohistochemistry (IHC)1+ or 2+ without amplification by fluorescent in situ hybridization (FISH), are considered HER2 negative and are not sensitive to standard anti-HER2 therapies. However, antibody-drug conjugates, delivering a potent payload though a “trojan horse” mechanism, could be effective in cancers with lower levels of HER2 expression and no strong addiction to HER2 signaling for cancer proliferation. Additionally, the membrane permeability and cytotoxic by-stander effect of novel payloads could also play a role in the antitumoral activity of ADCs in this setting [35]. In fact, T-DXd therapy led to clinical responses in the HER2-low BC cohort of the phase Ib study. Overall, in 54 heavily pretreated patients (median 7.5 prior lines) treated at a dose of 5.4 or 6.4 mg/kg, the ORR confirmed by independent review was 37% (95% CI, 24.3% to 51.3%) with median duration of response of 10.4 months (95% CI, 8.8 months to not evaluable) and median PFS of 11.1 months (95% CI, 7.6 months to not evaluable) [36]. A similar response rate has been observed in patients treated with SYD985 where patients with HER2-low MBC, including hormone-receptor positive (*N* = 32) and hormone-receptor negative breast cancers (*N* = 17) had ORRs of 27% and 40%, respectively [37]. Despite the limited sample size of these two cohorts, a signal of activity was observed in heavily pretreated HER2-negative patients with refractory disease. Based on the expanding potential for the new generation of HER2-ADCs in HER2-low expressing cancers, new methods to optimally define different thresholds of HER2 expression are needed to predict response to these therapies.

## ADCs for central nervous system (CNS)

Approximately 50% of HER2-positive MBC patients will develop brain metastases (BM) [38]. This confers an overall worse prognosis with shorter median OS compared to those without BM, 26.3 months versus 44.6 months, respectively [39]. Although ADCs have high molecular weights, evidence for clinical benefit of T-DM1 and T-DXd in HER2-positive BC patients with BM has been reported, suggesting permeability of these agents (or their payloads) across the blood-brain barrier. In the subgroup analysis of TH3RESA trial including 67 patients with stable BM at baseline, the median OS was 17.3 months for patients who received T-DM1 vs 12.6 months for those who received treatment of physician's choice (HR 0·62 [95% CI 0.34–1.13]) [9]. A more direct test of the CNS benefit of T-DM1 was reported in the KAMILLA trial in which the ORR in CNS metastases for patients without prior brain radiation was 49.3% (33/67; 95% CI 36.9–61.8) and the median duration of exposure to T-DM1 for the patients with brain metastases was 9.5 months (range 0.8–43.5) [40]. In contrast with these findings, T-DM1 seems to be not as effective in preventing CNS relapse in the early stage, as shown in the KATHERINE trial, where the incidence of brain metastases was similar in patients treated with adjuvant T-DM1 or trastuzumab (5.9 versus 4.3, respectively) [10]. In the phase II pivotal trial of T-DXd, the PFS of patients with treated and asymptomatic BM (*N*=24) was 18.1 months (95% CI, 6.7 to 18.1) versus 16.4 months for the overall population [15], again suggesting the potential for this ADC to have CNS activity. Ongoing trials specifically enrolling patients with active BM will clarify the role of T-DXd in this setting (Table 1). Recently, the newly approved HER2-targeted tyrosine kinase inhibitors tucatinib and neratinib have shown significant clinical benefit in the treatment of stable as well as progressive or untreated BMs, although no direct comparison with the new ADCs is yet available [41, 42]. Among patients with BM, patients with HER2-positive MBC have a longer median OS after the development of BM compared with luminal-like and triple-negative MBC (about 12 vs 6 months), which is postulated to be related to the CNS benefits of systemic HER2-targeted therapies [43]. Indeed, none of the regimens currently approved for the management of HER2-negative breast cancers has shown a significant impact on BM outcomes. Since newer HER2 ADCs are potentially active in HER2-low expressing breast cancers, their availability could offer some benefit for CNS disease in this high-risk population of patients and further study is warranted.

## Toxicity profile

The toxicity profile of ADCs appears to be correlated with the stability of the conjugates in the bloodstream and to the off-target effects of the payloads. Cardiotoxicity, mainly related to the mAb component, seems to be less of an issue with the newer ADCs compared to trastuzumab; however, selection bias against patients who experienced cardiotoxicity with trastuzumab may partially explain this finding. A recent pooled analysis of T-DM1 trials in the metastatic setting revealed a risk of congestive heart failure to be < 1%. Age ≥ 65 (OR 3.0; 95% CI, 1.77–5.14; *p* value < 0.001) and baseline LVEF < 55% (OR 2.62; 95% CI, 1.29–5.32; *p* value 0.008) were statistically associated with higher risk to develop any cardiac event [44]. In the ATEMPT study, which evaluated the activity of T-DM1 in patients with low-risk breast cancer who were naïve to HER2-targeted therapy, the rate of LVEF decline was consistent with data reported in advanced stage (heart failure: 0.8%, LVEF drop: 0.5%) [11]. Grade 3/4 LVEF declines have been reported with T-DXd and SYD985 at less than 1%.

Interstitial lung disease (ILD) is a risk associated with T-DXd therapy that requires vigilant monitoring of patients for respiratory signs and symptoms and for early intervention (including suspension of treatment and initiation of steroids) to prevent serious consequences. DESTINY-Breast01 investigators reported cases of ILD of any grade in 28/184 (15.2%) patients, as confirmed by an independent adjudication committee [16]. While most cases were grade 1 and 2 events that were reversible, there were 5 fatal cases of ILD attributed to T-DXd treatment. Across multiple tumor types including breast cancer, only 22/44 ILD cases reported by investigators have been judged as drug-related by the adjudication committee, revealing the difficulty in making this diagnosis [45]. The frequency of ILD reported in a pooled analysis evaluating T-Dxd in several malignancies, presented during the virtual AACR Annual Meeting 2021, is similar to that previously reported and the all-grade risk of ILD was observed to decrease after 12 months of treatment [46]. According to the FDA label, in patients with grade 2 or higher ILD, the treatment should be permanently discontinued, and any respiratory symptoms should be promptly investigated. The pathophysiology of the T-DXd-related lung damage is not well understood. An in vivo study conducted in cynomolgus monkeys injected with T-DXd at different doses revealed histopathological lung features of diffuse lymphocytic infiltrates and slight fibrosis with an incidence that was dose-dependent and dose-frequency-dependent. Immunohistochemical analysis further confirmed that T-DXd localization was mainly in alveolar macrophages, but not pulmonary epithelial cells suggesting a possible mechanism of lung injury related to target-independent uptake of T-DXd into alveolar macrophages [47]. Additional work will be necessary to clarify these findings and their clinical implications.

Ophthalmic toxicity has been considered a class-related toxicity and includes a large spectrum of ocular adverse events, usually of low grade and reversible, including conjunctivitis/keratoconjunctivitis, uveitis, corneal epithelial damage, and optic neuropathy [48]. Other ADC-related adverse events include bone marrow toxicity, nausea, fatigue, vomiting, and peripheral neuropathy, occurring in different frequencies and largely attributed to the specific payload. Adverse events of grade 3 or greater reported on clinical trials with frequency of at least 2% and special class-related toxicities of ADCs are listed in Tables 3 and 4, respectively.

**Table 3:** Anti-HER2 ADCs treatment-related grade ≥ 3 adverse events occurring in > 2% of patients

Toxic effect	ADCs	Frequency	Possible mechanism
Thrombocytopenia	T-DM1 [7] T-DXd [15]	14.3% 4.3%	DM1-induced impairment of megakaryocytes differentiation Bone marrow toxicity of payload
Neutropenia	T-DM1 [7] T-DXd [15] SYD985 [18] RC48-ADC [23] ALT-P7 [24]	2.4% 20% 14% 10% 14%	Bone marrow toxicity of payloads
Anemia	T-DM1 [7] T-DXd [15]	3.8% 8.6%	Bone marrow toxicity of payloads
Leukopenia	RC48-ADC [23]	6.7%	Bone marrow toxicity of payloads
Decreased lymphocyte count	T-DXd [15]	8.1%	Bone marrow toxicity of payloads
Fatigue	T-DM1 [7] T-DXd [15] SYD985 [18] PF-06804103 [28]	2.4% 6% 3% 5.7%	Unknown
Arthralgia	PF-06804103 [28]	5.7%	Unknown
Increased ALT Increased ALT	T-DM1 [7] RC48-ADC [23] RC48-ADC [23]	15% 3.3% 3.3%	Cytotoxic activity on the hepatocytes (clearance of T-DM1 depends mainly on the hepatobiliary and gastrointestinal route)
Nausea	T-DXd [15]	7.6%	Off-target effect of the payload
Vomiting	T-DXd [15]	4.3%	Off-target effect of the payload
Diarrhea	T-DXd [15]	2.7%	Off-target effect of the payload
Peripheral neuropathy	T-DM1 [49] PF-06804103 [28]	13% 5.7%	Degeneration of the axons, time-dependent, due to the DM1 component Systemic release of payload

This table includes all the ADCs discussed in this review (approved and under development) for which toxicity data were available at the time of the last editing (April 2021)

**Table 4:** Anti-HER2 ADC toxicities of special interest

Toxic effect	ADCs	Frequency	Possible mechanism
Interstitial lung disease Pneumonitis	T-Dxd [46] ARX788 [25]	Any grade: 15.5% Grade 3:1% Grade 4: 0.1% Grade 5: 2.4% Any grade: 19.6% Grade 3: 2%	Alveolar inflammation and fibrosis due to off-target activity of DXd Unknown
LVEF drop	T-DM1 [44] T-Dxd [15]	Any grade: 2% Grade 3–4: 0.7% Any grade: 1.6% Grade 3–4: 0.5%	Target-related: morphologic and functional potentially reversible damage to cardiomyocytes inducted by anti HER2-Ab
Prolonged QT	T-Dxd [15]	Any grade: 4.9% Grade 3–4: 1.1%	Unknown
Ocular toxicities	SYD985 [18] A166 [19] ARX788 [25]	Conjunctivitis All grades: 31% Grade 3/4: 3% Keratitis: All grades: 19% Grade 3/4: 2% Overall and any grade: 80% Overall and any grade: 41.2 %	Systemic releasing of the payload

All patients included in these trials received prior anti-HER2 therapies in metastatic and/or adjuvant setting

## Conclusions

ADCs development has been one of the most successful advances in breast oncology in the last decade. The innovative molecular structure, combining antigen specificity with potent cytotoxic effects, confers unique pharmacodynamic and pharmacokinetic properties to ADC therapy. The efficacy of next-generation ADCs in HER2-low tumors has the potential to change the historical paradigm of HER2-addiction and response to HER2-targeted therapy. New strategies to define HER2 status are needed in order to identify patients that are most likely to derive clinical benefit. In the wake of the success of T-DM1 and T-DXd, a plethora of new anti-HER2 ADCs with promising preclinical and early clinical activity are currently under investigation. With the potential to offer a favorable therapeutic index even in heavily pretreated patients, there is great hope that these agents will soon be incorporated in early lines and early disease settings. There remain questions regarding the mechanism of action and specific mechanisms of organ injury that are yet to be elucidated and this is critical in order to safely expand the use of these therapies to a broader group of patients.

## Data Availability

We developed a research strategy run on PubMed using research terms “anti-HER2 antibody drug conjugate,” “metastatic HER2- positive breast cancer,” “breast cancer,” and “antibody drug conjugate,” variously combined (no timing and language restriction; first run on January 31, 2021; last run: April 8, 2021). The abstract submitted to ASCO, ESMO, AACR, and SABCS meeting between 2018 and 2020 and AACR meeting 2021 were manually searched. Additionally, we used this tool “https://www.adcreview.com/adc-drugmap/,” designed to collect ADCs under development for any malignancy: breast cancer filter was entered, and anti-HER2 ADCs were selected manually. Eventually, to identify the ongoing trials, we consulted ClinicalTrial.gov.

## Abbreviations

AACR: American Association for Cancer Research
ADCs: Antibody-drug conjugates
ADCC: Antibody-dependent cellular cytotoxicity
anti-PD1: Anti programmed cell death protein 1
ASCO: American Society of Clinical Oncology
BM: Brain metastases
CR: Complete response
DAR: Drug to antibody ratio
DCR: Disease-control rate
DOR: Duration of response
ESMO: European Society of Medical Oncology
Fab: Fragment antigen-binding
Fc: Fragment crystallizable
FDA: Food and Drug Administration
FISH: Fluorescent in situ hybridization
HER2: Human epidermal growth factor receptor 2
IDFS: Invasive disease-free survival
IgG: Immunoglobulin G
IHC: Immunohistochemistry
ILD: Interstitial lung disease
mAB: Monoclonal antibody
MBC: Metastatic breast cancer
MMAE: Monomethyl auristatin E
ORR: Objective response rate
OS: Overall survival
PARP: Poly ADP-ribose polymerase
pCR: Pathological compete response
PFS: Progression-free survival
PR: Partial response
SABCS: San Antonio Breast Cancer Symposium
SD: Stable disease
